# Stability of similarity measurements for bipartite networks

**DOI:** 10.1038/srep18653

**Published:** 2016-01-04

**Authors:** Jian-Guo Liu, Lei Hou, Xue Pan, Qiang Guo, Tao Zhou

**Affiliations:** 1 Data Science and Cloud Service Research Centre, Shanghai University of Finance and Economics, Shanghai 200433, PR China; 2 Research Center of Complex Systems Science, University of Shanghai for Science and Technology, Shanghai 200093, PR China; 3 Informatics Research Center, Henley Business School, University of Reading, Whiteknights, RG6 6UD, United Kingdom; 4 Web Sciences Center, University of Electronic Science and Technology of China, Chengdu 610054, PR China

## Abstract

Similarity is a fundamental measure in network analyses and machine learning algorithms, with wide applications ranging from personalized recommendation to socio-economic dynamics. We argue that an effective similarity measurement should guarantee the stability even under some information loss. With six bipartite networks, we investigate the stabilities of fifteen similarity measurements by comparing the similarity matrixes of two data samples which are randomly divided from original data sets. Results show that, the fifteen measurements can be well classified into three clusters according to their stabilities, and measurements in the same cluster have similar mathematical definitions. In addition, we develop a top-*n*-stability method for personalized recommendation, and find that the unstable similarities would recommend false information to users, and the performance of recommendation would be largely improved by using stable similarity measurements. This work provides a novel dimension to analyze and evaluate similarity measurements, which can further find applications in link prediction, personalized recommendation, clustering algorithms, community detection and so on.

Connections are everywhere and can be observed between everything in our world^1^,^2^, such as the connections between the online users and their rated or selected objects^3^,^4^ and the connections between neurons in the neural networks^5^,^6^. To characterize systems consisting of connections in which objects are represented as nodes and connections are represented as links, complex networks have been widely used in the last decade to study the relations between different objects and the structure of those kinds of systems^7^,^8^,^9^.

However, in most real complex networks, every pair of nodes has some specific relations even there is no link between them. For example, citation networks consist of papers. If one paper cited another, there would be a link between them^10^. Besides citing relations, other potential relations may exist when the papers have same authors or cited same papers and so on^11^. Generally, *similarity* describes the connections between different objects’ properties, which is the most used method to evaluate such relations. And the similarity has become an important measurement with great significance for both theoretical researches (such as biological and physical science) and practical applications (such as the e-commerce and social service). For example, in biological analysis, evaluating the similarity of genes’ expression profile, one may identify similar regulations and the control processes of genes^12^,^13^. Co-expression networks may also be established according to the similarities between genes^14^,^15^. While protein-protein or metabolic interactions can only be verified by costly experiments and most of the interactions are still unknown, similarity-based link prediction method^16^,^17^ could largely help identify the most likely pair of interacting proteins^18^,^19^,^20^. In addition, the similarity measurements have also found its applications in object clustering^21^, community detecting^22^,^23^, machine learning^24^,^25^ and socio-economic dynamics^26^. As to more practical applications, in term of the similarities, recommendation systems^27^,^28^ evaluate correlations between objects such as movies, commodities, books and so on, and accordingly make recommendations to users.

So far, dozens of similarity measurements have been developed. However, with different data, similarity measurements generally have very different performances. Even with different parts of a same data, the results may be also different. Particularly in the bipartite networks, the object similarity is determined by their natural properties, and thus, similarity should be steadfast for a definite pair of objects. On the other hand, the networks we investigated are mapped incompletely, which is always evolving, or contain false positives and negatives^29^. In the user-object bipartite networks, since the natural properties of the objects are unchangeable, a good similarity measurement should always return the same values for each pair of objects. To explore the stability problem of object similarity for bipartite networks, fifteen similarity measurements will be analyzed and studied in this paper. Firstly, we will report the influence of data amount on the stabilities of fifteen similarity measurements. Secondly, the comparison and classification of the fifteen similarity measurements will be analyzed. Finally, we will explore the effect of the object similarity stability on the recommendation.

## Similarity Measurements

In many online systems, objects usually could get ratings from different users. To this kind of context, one can use the *Cosine Index* (COS) or *Pearson Coefficient* (PC) to measure the object similarity. When the ratings are unavailable, similarity can also be defined from the structure of the historical data, that is, two objects are similar if they are connected with many same users. The simplest such method is *Common Neighbour* (CN), where the similarity between two objects are directly given by the number of same neighbours who have connections with them. Considering the degree information of two objects, variations of the CN index have been proposed, including the *Salton Index* (SAL)^30^, *Jaccard Index* (JAC)^31^, *Sørensen Index* (SOR)^32^, *Hub Promoted Index* (HPI)^33^, *Hub Depressed Index* (HDI) and *Leicht-Holme-Newman Index* (LHN)^34^ indices. Instead of the number of the same neighbours in the CN index, the *Adamic-Adar Index* (AA)^35^ and *Resource Allocation Index* (RA)^36^ indices were presented, regarding the object similarity as the summation of their common neighbours’ degrees. According to the preferential attachment process^8^, the *Preferential Attachment Index* (PA) was also presented. Furthermore, using the concepts from physics, the *Mass Diffusion* (MD)^37^, *Heat Conduction* (HC)^38^,^39^ and *Improved Heat Conduction* (IHC)^40^ methods were also investigated. The mathematical definitions of those similarity measurements can be found in the Method Section. Generally speaking, the value of similarity is relatively high (low) if the objects are very similar (different). With these fifteen similarity measurements, we investigate the similarity stability for the user-object bipartite networks.

## Data

Six different data sets are applied in this paper to study the stability of similarity measurements, differing both in the subject matter and data sparsity, as shown in Table 1. These data sets are usually modelled as the user-object bipartite networks and widely used to investigate the performance of the recommendation algorithms^41^,^42^,^43^. The *MovieLens* and *Netflix* data sets are movie Web Sites in which users could watch and rate movies. The *Amazon* data set is an e-commerce system in which users buy commodities. The *Last.FM* data set is a music Web site allowing users to collect different artists’ music. The *Epinions* data set allows users writing reviews and on the other hand reading others’ reviews. The *Del.icio.us* data set is a bookmark Web Site in which users collect and share bookmarks they interested in.

## Similarity Stability

**Figure 1:**
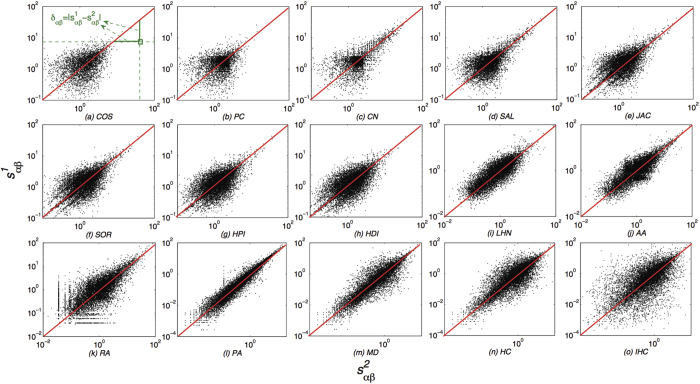
Typical examples of the comparison between object similarities 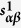 and 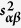 of the MovieLens data set for fifteen similarity measurements. When dividing the data set, we set 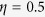, i.e. 50% ratings are divided into the first sample and the others are divided into the second one. For each calculation, we randomly select 10^4^ pairs of objects’ similarities of two samples to show in the figure. Thus, there are 10^4^ dots in each subplot, each represents a pair of objects. The dots would locate on the diagonal if the similarities in two samples 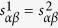. Consequently, the more stable the similarity measurement is, the more concentrated the dots would distribute around the diagonal.

Although lots of object similarity measurements have been presented, we could not know the exact object similarity. Thus, to examine the stability of those measurements, we divide the data set into two samples to compare the similarity matrixes calculated from those two samples for each measurement. The data-dividing method can be described as follow: Every record will get a random number *p* from a uniform distribution ranging from 0 to 1, and this record belongs to the first sample if 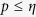 and belongs to the second sample if 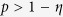, where 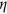 can be regarded as a data amount parameter and 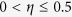. With this method, those two samples would have no overlaps, which means, they are totally different parts of the data set. For a specific pair of objects *α* and *β*, we use 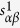 to denote their similarity in the first sample and 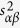 to denote that in the second sample. Thus, if a similarity measurement can give stable evaluation of the object similarity, there would be 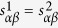. Figure 1 reports the distributions of similarities of two samples for each of the fifteen similarity measurements in MovieLens data set. The dots would distribute near the diagonal if the measurement can give stable evaluation of object similarity. The PA index presents the most concentrated distribution. The reason lies in the fact that the PA index only considers the neighbour node popularity. Popular objects of a data sample are in general also popular in another data sample, and thus the object similarity is stable. Other measurements’ results are not so concentrated especially for pairs of objects with low-similarity pairs of objects. Results in Fig. 1 indicate that, when the data is changed, a same pair of objects may be evaluated as different similarity levels and thus, the stability problem indeed exists in most similarity measurements.

The similarity values calculated by different measurements distribute in different ranges, and thus we make a simple normalization to compare the measurement stability. Suppose that 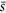 is the average value of similarities that 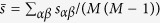 where *M* is the number of objects which have at least one record in the corresponding sample, the normalization is 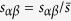. Specifically, the similarities of the PC index distribute in the range 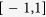, which may probably leads 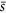 to be 0. Hence, we make the normalization as 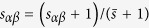 for the PC index. Henceforth, the similarities are all been normalized before used. To qualify the stability of object similarity, we define three metrics:

1. The *average bias μ* is used to describe the average level of similarity difference between two similarity matrixes from two samples, and it reads
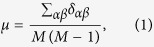
where 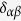 is the bias of similarities between objects *α* and *β* from two samples as shown in Fig. 1(a), i.e. 
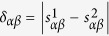
. High value of average bias means, on average, the same pair of objects is evaluated as different similarities when the data is changed. Therefore, the more stable the similarity measurement is, the lower value *μ* would be.
2. The *standard deviation of bias σ* reads
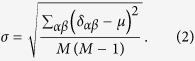
The deviation *σ* can measure the difference of susceptibility between different pairs of objects against the data change. High values of the deviation *σ* mean that similarities between some pairs of objects may be quite unstable. On the other hand, low values of *σ* indicates that, each pair of objects has similar unstable level and the bias *μ* may be caused by the coincident entirety changes of each pair of object similarities.
3. The *Pearson coefficient ρ* reads

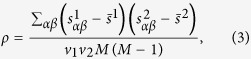


where 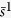 and 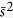 are the average value of similarity over every pair of objects for two samples respectively, and 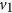 and 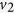 are the standard variance of similarities for two samples respectively. In general, the value of Pearson coefficient *ρ* ranging from −1 to 1 measures the coherence of two similarity matrixes calculated by two samples. The upper limit of Pearson coefficient *ρ* = 1 means two similarity matrixes are totally coherent and the corresponding similarity measurement is totally stable.

**Figure 2:**
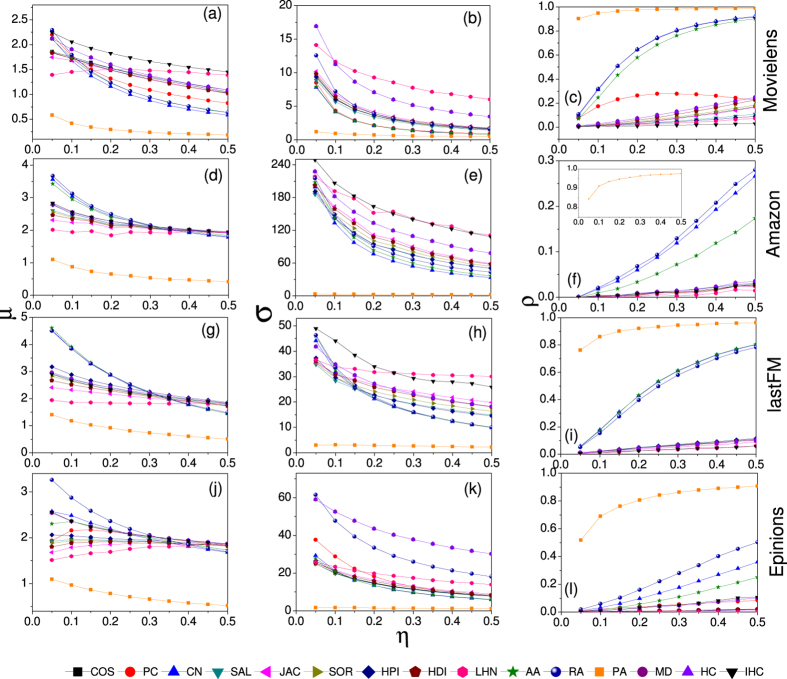
Average bias *μ*, standard deviation of bias *σ* and the Pearson coefficient *ρ* against the data amount parameter *η* for the MovieLens, Amazon, Last.FM and Epinions data sets respectively. Each data point is averaged over 20 independent experiments, i.e., for each level of data amount parameter *η*, we randomly divide the data for 20 times and calculate *μ, σ* and *ρ* of each time. Note that, there is only selecting information without ratings in the Last.FM data set. Thus, the COS and PC indices could not be performed in the Last.FM data set. As the data becomes more and more abundant, the stability of object similarity would relatively be better. However, many measurements still could not give stable evaluation of object similarity.

For each similarity measurement, we calculate the similarity for two data samples with different data amount parameter 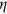. The results of average bias *μ*, standard deviation of bias *σ* and the Pearson coefficient *ρ* of the MovieLens, Amazon, Last.FM and Epinions data sets are reported in Fig. 2 (Results of Netflix and Del.icio.us datasets can be found in the Supplementary Information). One can easily find that, the PA index is the most stable measurement regardless of the data amount 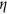. Even with small size of data, the PA index could give stable evaluation of the object similarity. As the data amount increases, the average biases *μ* and the standard deviations of bias *σ* generally decrease. It can be observed that for both the average bias *μ* and deviation *σ*, the CN, AA and RA indices have similar decay patterns. When the data amount is small 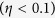 the average bias *μ* of the CN, AA, RA indices are almost the highest, and with the increase of 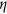, the average bias *μ* rapidly decreases which means they are sensitive to the data amount. Another dynamic cluster consisting of the COS, SAL, JAC, SOR, HPI, HDI indices seem to be insensitive. Although the average bias *μ* and deviation *σ* also decrease with the increment of data, the decays are much slower than that of the former cluster (the CN, AA, RA indices). A special measurement refers to the LHN index, which has no apparent dynamic against the data amount 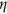. Same with the results of average bias *μ* and deviation *σ*, the Pearson coefficient *ρ* of the PA index is the highest and larger than 0.9 even with the smallest data amount 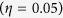. As to the CN, AA, RA indices, the Pearson coefficients *ρ* are also sensitive, which is similar to the average bias *μ* and deviation *σ*. As the data amount 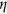 increases, the Pearson coefficient *ρ* of the CN, AA, RA indices rapidly increase to quite high levels. Other measurements’ Pearson coefficients *ρ*, however, increase very slowly with the data amount and are in general less than 0.2 even when all the data 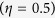 is used. Especially in Amazon which is a very sparse (sparsity is 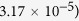 data set, the Pearson coefficients *ρ* of most measurements are less than 0.03. This result indicates that, for most similarity measurements, the similarity matrixes calculated from different data samples could have no apparent coherence. Overall, more data could make it more stable for most of the measurements especially the CN, AA and RA indices.

**Figure 3:**
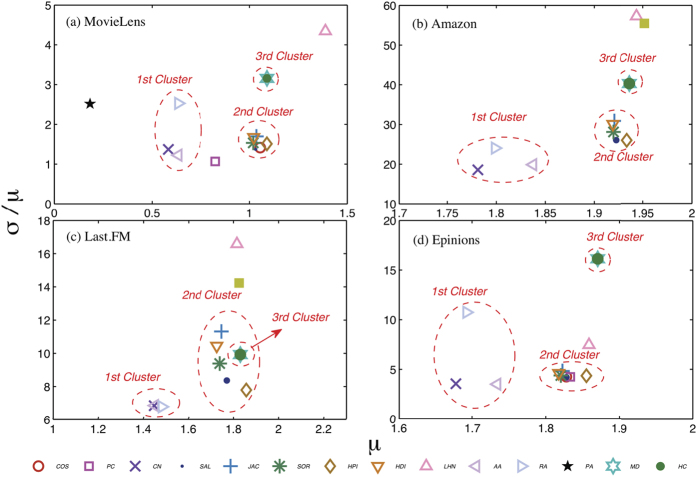
The *μ*–*σ* location map with data amount parameter *η* = 0.5. On the location map, a measurement locating on the left side means the similarities of objects have little change at average, and the bottom means the similarities of each pair of objects have similar changes. Overall, a stable measurement generally would locate on the left bottom of the 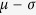 location map.

To get deeper insight of the comparison and the classification of these similarity measurements, we analyze the results of the average bias *μ* and standard deviation *σ* when all of the data is used 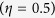 which is shown in Fig. 3 (Results of the Netflix and Del.icio.us data sets can be found in the Supplementary Information). Using the average bias *μ* and dispersion 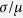 as two dimensions, we can get the 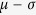 location map for each data set. Surprisingly, one can find that, these fifteen similarity measurements could be well classified from the perspective of similarity stability. Except four measurements namely the PA, PC, LHN and IHC indices, the others could be classified into three clusters. Measurements in the same cluster are similar in both mathematical forms and original considerations. The first cluster consists three measurements namely the CN, AA and RA indices, all of which only take into account the information of common neighbours of two target objects. Besides the CN index which considers the number of common neighbours, the AA and RA indices calculate the total number weighted by 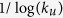 and 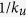 respectively where 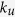 is the degree of the common neighbour *u* of the two target objects. The second cluster consists of six measurements namely the COS, SAL, JAC, SOR, HPI and HDI indices. Except the COS index, the other five measurements are all variations of the CN index. However, another variation of the CN index, namely the LHN index, locates outside the second cluster. The reason may be that, when considering the degree information of two target objects, the LHN index makes the degrees of two objects multiplied, i.e. 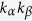, thus the degree information is quadratic in the LHN index. Unlike the LHN index, other variations’ degree information is not quadratic, such as 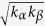 of the SAL index, 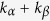 of the SOR index, 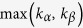 of the HDI index and so on (See the Method Section for detailed mathematical definitions of these measurements). The third cluster consists the MD and HC indices which consider the degree information of both the target objects and their common neighbours. Another similar point is that, both the MD and HC indices are designed based on the spreading process on bipartite networks. Although the basic considerations are different, mathematical definitions of the MD and HC indices are very similar which leads to 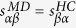. Overall, according to the stability of the object similarity, various similarity measurements could be well classified into three clusters. In fact, the classification can also be observed in Fig. 2, in which measurements in the same cluster always have same dynamical patterns against the data amount parameter 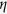.

## Results on the Artificial Data

To explore whether the stability pattern is due to the property of the data set or the nature of each measurement, we present two methods to test the similarity stability on the reshuffled and randomly generated data sets. 1) In the first method, we reshuffle the links between users and objects from the empirical data sets. At each step, we randomly select two records, say 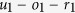 and 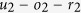, and exchange two objects 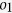 and 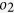 or two ratings 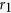 and 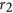 with equal probabilities. After enough steps, the data set would be reshuffled greatly. In our case, we perform 10*T* steps for reshuffles, where *T* is the number of records in the corresponding empirical data set. 2) The second method is randomly generated data sets. Initially, we suppose there is an empty bipartite network with *M* objects and *N* users. Then we randomly generate *T* links between users and objects. At each step, we randomly select an object and a user, where the selection probabilities of each object and user are 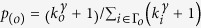 and 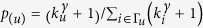 respectively, where 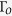 and 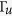 are the sets of objects and of users. Furthermore, considering the COS and PC measurements, we randomly generate an integer rating ranging from 1 to 5 for each record. In our study, we have 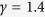. Under this scheme, after enough steps, the bipartite network would emerge power-law degree distributions for both users and objects. Note that, with fixed *M* and *N*, the number of links *T* could control the average degree of the bipartite network, say, 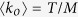 and 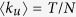.

**Figure 4:**
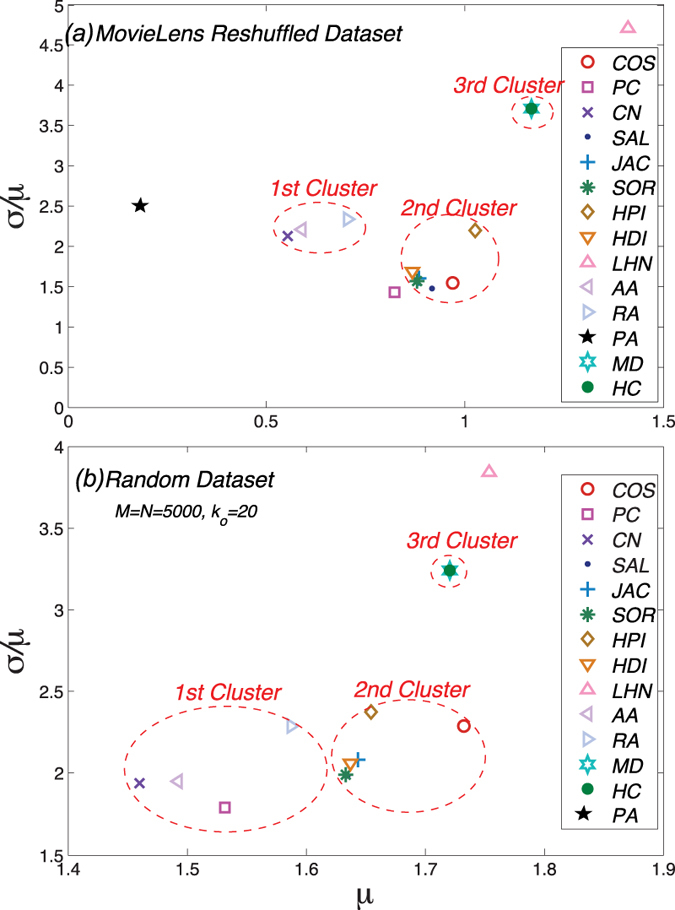
The *μ*–*σ* location map for (**a**) reshuffled MovieLens data set with parameter *η* = 0.5 and (**b**) randomly generated data set with *M* = *N* = 5000 and 〈*k*_*o*_〉 = 20. On each map, we still mark the clusters we observed in the empirical results which are shown in Fig. 3. It turns out that, measurements in the same clusters still have similar similarity stabilities even when the user behavior patterns are removed. Thus, the similarity stability patterns are due to the nature of each measurement, not the properties of the data sets.

For the reshuffled and randomly generated data sets, we perform the same calculations used for the empirical data sets. Figure 4 reports the 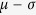 location maps of the data sets. The subplot (a) shows the location map of reshuffled data set. Taking the MovieLens data set as an example, the measurements classified into the same cluster are still in the same area on the 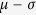 location map. The reshuffle process does not change the stability pattern of each measurement. Actually, the measurements in the same cluster have similar stability trends against the data amount parameter 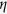 which is shown in Fig. S7 of the Supplementary Information. For the randomly generated data set, the subplot (b) shows the location map with parameter 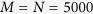 and 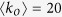. Although the differences may be larger than the empirical results, the locations of measurements in the same cluster are still close to each other. Since the structure of the randomly generated data set could be controlled, we explore the effect of the structure influence on the similarity stability, and the results are shown in the Supplementary Information. In summary, the results of the reshuffled and randomly generated data sets suggest that the stability patterns come from the nature of each measurement.

## Effect on the Recommendation

Object similarities of the user-object bipartite networks are generally used for recommendations^3^. Although the fifteen similarity measurements are widely used in the recommendation systems, the stability of the recommendation regarding to the similarity measurements is still unknown. In this section, we analyze the effect of object similarity stability on the recommendation results. Generally speaking, the goal of a recommendation system is to generate a recommendation list of *L* objects and voluntarily to display on each user’s interface based on the target user’s historical selections. The system predict the scores for every unselected objects to a target user *u*, and rank the objects from high scores to low ones. The score of an object *β* for the target user *u*, 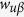, is given by


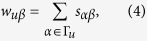


where 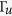 is the object set which consists with the historical selections of the user *u*. A high score means that, the system evaluates it as what the target user interests in. To quantify the stability of recommendation results, we divide the data sets as two samples according to the former method with 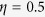. For a target user *u*, there would be two ranking lists of objects. If an object *α* is ranked at the *i*th position of the ranking list, we define 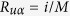 as the ranking score where *i* is object *α*’s position in another ranking list and *M* is the number of objects. Hence, we can use the average ranking position 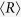 to describe the stability of the recommendation results and 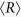 reads


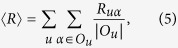


where 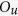 is the object set ranking at the top *L* positions of the ranking list and at the same time have not been selected by the target user *u* in both of the samples, and 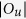 is the number of objects in the set 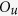. According to this definition, stable measurements would have small average ranking position 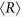.

According to Eq. (5), we calculate the recommendation stability and find that, many of the recommendations are quite unstable (Table S1). The average ranking position 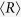 of SAL, HPI, LHC, HC and IHC indices are even larger than 0.1 in each data set. Taking the MovieLens data set as an example, 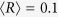 means that, when using the similarities of another sample data, the objects recommended at the top *L* positions using a data sample are ranked at 585th position (there are 5850 objects in the MovieLens data set). Theoretically, the average ranking position 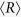 of the totally random case is 0.5 and the most stable results is 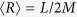 where *M* is the number of the objects. Thus, the theoretical best stability is 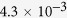, 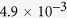, 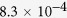 and 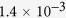 for the MovieLens, Netflix, Epinions and Last.FM data sets respectively, which means the recommendation lists of the two data samples are very close. Furthermore, if compare the similarity stability *ρ* with the recommendation stability 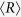, one may find that, the more unstable the similarity quantification is, the more unstable the recommendation generally would be (Fig. S3).

To improve the stability of the recommendation and explore the effect of the similarity stability, here we present a top-*n*-stability method. For an object *α*, the similarity bias 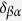 between object *α* and *β*, is calculated and ranked from the lowest value (stable) to the highest one (unstable). According to Eq. (4), when adding the score of object *α*, we only take *n* objects which have the most stable similarities i.e., which ranks at the top *n* positions to object *α*. This could be explained as


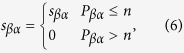


where 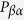 is the position of object *β* in object *α*’s stability list. Note that, unlike the classical top-*n*-similarity recommendation algorithm in which *n* objects with the highest similarities to object *α* would be counted^44^,^45^, here we ignore the exact value of similarity, just consider the stability. The basic consideration is that, if one pair of objects’ similarity has poor stability, the similarity would be meaningless regardless of the value of similarity. Through the experiments, the classical top-*n*-similarity method can also improve the recommendation’s stability for a little bit, but the improvement of our top-*n*-stability method is much bigger (See the Supplementary Information).

**Figure 5:**
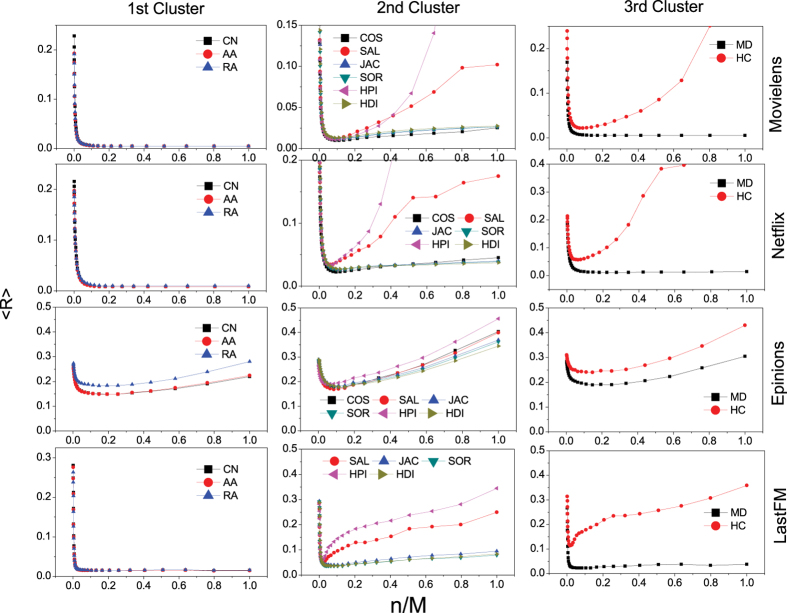
The average ranking position〈*R*〉of the recommended objects, against number of objects that counted in the top-*n*-stability method. The length of the recommendation list in the simulation is *L* = 50, and the results are averaged over 10 independent simulations. In general, the recommendation stability could be improved by considering only the stable similarities.

With different number of stable objects *n*, Fig. 5 shows the average ranking position of the recommended objects 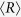, which is summarised according to the similarity measurement clusters. The results of similarity measurements the PC, LHN, PA and IHC indices could be found in the Supplementary Information. One can find that, there is no apparent recommendation stability improvement for the first cluster (the CN, AA, RA indexes) except in the Epinions data set in which the recommendation stability is poor for every similarity measurement. On the other hand, recommendation stability of measurements of the second cluster could be well improved by the top-*n*-stability method especially for the SAL and HPI indices whose average ranking position 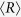 are over 0.1. However, measurements in the third cluster, i.e. the MD and HC indices, have different patterns against the top-*n*-stability method. Although the HC index’s recommendation stability could be largely improved, the MD index has no apparent improvement. We can conclude that, when the recommendation is unstable, our top-*n*-stability method could largely improve (See Table S2 for detailed improvement ratio) the stability by taking only the stable similarities into account. For most similarity measurements, when considering around 10% of the similarities, the optimized stability could be reached. And for the poor-stability measurements, the counted ratio may even be about 5%. The improvement indicates that, those unstable similarities are more like false information which would lead to the deflected evaluation of users’ true interests.

## Conclusion and Discussions

The similarity measurements can evaluate the potential relations between objects in the biological, social, commerce systems, they are meaningful only if the evaluated similarities are stable when the nature of the objects are unchangeable. Unstable similarities are generally false information which would lead to the misunderstanding of the relations between objects. We investigated the stabilities of fifteen similarity measurements for user-object bipartite networks, and found that when measuring the object similarity, most similarity measurements except the PA, CN, AA, RA indices, are quite unstable. The Pearson coefficient *ρ* of two similarity matrixes calculated from two data samples may be even smaller than 0.2, which means the two matrixes have little correlation. Generally speaking, measurements with simple considerations can describe the natural properties of objects and are stable. The CN, AA, RA indices considering only the information of two objects’ common neighbours are stable and can be regarded as one cluster. On the other hand, variations of the CN index, namely the SAL, JAC, SOR, HPI, HDI indices, considering further the degree information of two objects, are less stable than the CN index and can be regarded as another cluster. Measurements in the same clusters have in general similar considerations and mathematical definitions, and thus have similar stabilities and even the dynamic against the data amount. In other words, while dozens of measurements have been developed, those similarity measurements can be well classified according to their object similarity stability. When a new measurement is proposed, one just need to analyze its stability to identify which cluster it belongs to, and then could get deeper insight to this measurement by comparing with other measurements within the same cluster. In addition, we presented a top-*n*-stability method to investigate the effect of object similarity on the recommendations. By considering only the stable similarities i.e. deleting the unstable, false information, the stability of the recommendation could be improved.

The investigations and considerations in this paper only focused on the objects. Actually, similarity is also an important method measuring the potential relations of human beings in the social systems and users in the online systems^2^,^46^. However, different with objects whose natural properties are definitely unchangeable, evidences have been found to prove that, the behaviors and interests of human behavior are temporal^4^,^47^. Thus, the stabilities of object similarity and human-to-human similarity may have totally different meanings. Additionally, the stability of those similarity measurements should be also studied in one-mode systems, which contain only one kind of nodes. Especially for the objects like genes, proteins etc., the investigations of similarity stability are still urgently needed because those objects may have different properties.

## Methods

The data sets used in the this paper are usually modelled as user-object bipartite networks in which nodes can be divided into two groups, representing users and objects respectively. In such kind of system, links only exist between different kinds of nods, i.e. between users and objects. We use *α* and *β* to denote the target pair of objects and 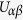 is the set of users who select both objects *α* and *β*. The popularity 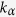 and 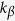 represent the selection times of object *α* by users respectively, and the activity 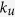 is the number of objects user *u* have selected. We suppose that, the function 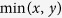 equals to the minimum value between *x* and *y* and 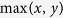 equals to the maximum value between *x* and *y*. In addition, 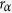 and 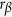 are rating vectors in the *N*-dimensional user space and 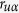 and 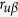 are the ratings user *u* given to the object *α* and *β* respectively. With these defined parameters, the fifteen similarity measurements referred in this paper read:


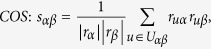



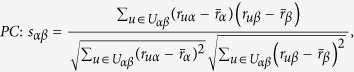



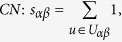



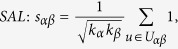



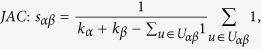



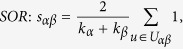



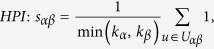



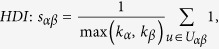



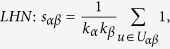



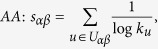



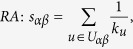



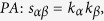



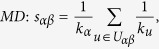



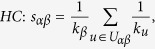



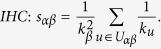


## Additional Information

**How to cite this article**: Liu, J.-G. *et al.* Stability of similarity measurements for bipartite networks. *Sci. Rep.* **6**, 18653; doi: 10.1038/srep18653 (2016).

## Supplementary Material

Supplementary Information

## Figures and Tables

**Table 1:** Properties of the utilized data sets.

Dataset	Subject matter	NUM. of users	NUM. of objects	NUM. of links	Sparsity
*MovieLens*	Movie	5547	5850	698054	2.15 × 10^−2^
*Netflix*	Movie	8608	5081	419247	9.59 × 10^−3^
*Amazon*	Commodity	645056	99622	2036091	3.17 × 10^−5^
*Last.FM*	Artist	1892	17632	92834	2.78 × 10^−3^
*Epinions*	Reviews	28090	30073	422085	5.00 × 10^−4^
*Del.cioi.us*	Bookmark	1861	1860	15328	4.43 × 10^−3^

The sparsity is the deviation between existed links and possible links, i.e. *T*/(*M* ⋅ *N*), where *T, M, N* are the number of links, objects and users respectively. Subject matters of those data sets are definite objects whose properties are unchangeable except Last.FM. The subjects of Last.FM are artists. However, the artists’ music have definite contains and properties. Thus, the artists in Last.FM could also be regarded as objects.
