# Supplementary material for: Stability of similarity measurements for bipartite networks
Source: Sci Rep. 2016 Jan 4;6:18653. doi: 10.1038/srep18653 (PMC4698667; doi:10.1038/srep18653)
Supplement: Supplementary Information [file srep18653-s1.pdf]

Stability of similarity measurements for bipartite networks

Supplementary Information

Jian-Guo Liu[1,2,*], Lei Hou[2,3], Xue Pan[2,3], Qiang Guo[2], and Tao Zhou[4]

1. Data Science and Cloud Service Research Centre,Shanghai University of Finance and Economics, Shanghai 200433, PR China.

2. Research Center of Complex Systems Science, University of Shanghai for Science and Technology, Shanghai 200093, P. R. China

3. Informatics Research Center, Henley Business School, University of Reading, Whiteknights, RG6 6UD, United Kingdom

4. Web Sciences Center, University of Electronic Science and Technology of China, Chengdu 610054, P. R. China

* liujg004@ustc.edu.cn

I. Similarity stabilities for the Netflix and Delicious datasets 2

II. The top-n-stability method 3

III. The top-n-similarity method 7

IV. Toy Dataset Test 8

I. Similarity stabilities for the Netflix and Delicious datasets

The present paper studies the similarity stability problem with six datasets with only four shown in the original paper. Here we report the results of the other two datasets, *Netflix* and *Del.icio.us*.

In the Netflix dataset, the dynamics of average bias μ, standard deviation of bias σ, and the Pearson coefficient ρ against the data amount parameter η are very similar with that of the other datasets. PA is the most stable index when measuring the object similarity. On the other hand, CN, AA and RA also show their well stabilities when the data amount is sufficient. Other indexes are somehow unstable, and their Pearson coefficients ρ are less than 0.2 even with all the data. However, the difference between each index in Del.icio.us dataset is not as apparent as in other datasets. While the Pearson coefficients of CN, AA, RA and PA in other datasets are much larger than other indexes that of all indexes are similar with each other. Unlike in other datasets, those unstable indexes such as LHN, HC and IHC also have Pearson coefficients larger than 0.2 in Del.icio.us dataset. This may be caused by the structure of Del.icio.us data.

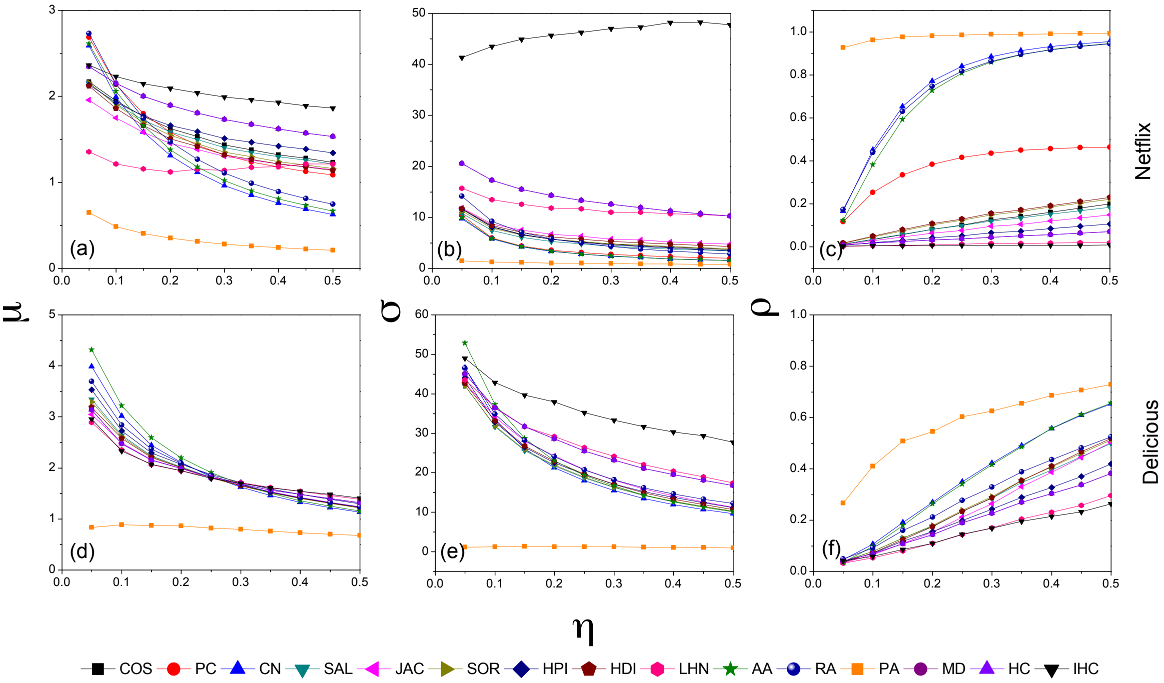

Fig. S1. The average bias μ, standard deviation of bias σ, and the Pearson coefficient ρ against the data amount parameter η for Netflix and Del.icio.us dataset.

As to the $\mu-\sigma$ location map, Netflix dataset exhibit the same classification of the similarity indexes with other datasets that, the first cluster consists of CN, AA, RA, the second cluster consists of COS, SAL, JAC, SOR, HPI and HDI, and the third cluster consists of MD and HC. However, in the Del.icio.us dataset's $\mu-\sigma$ location map, the RA index is located outside of the first cluster. Actually, one may find that, the locations of the indexes on the Del.icio.us dataset's $\mu-\sigma$ location map is very close to each other. Furthermore, many indexes also have similar Pearson coefficient in the Del.icio.us dataset, which is different with the other datasets. Overall, the classification still could be observed for the second and the third cluster.

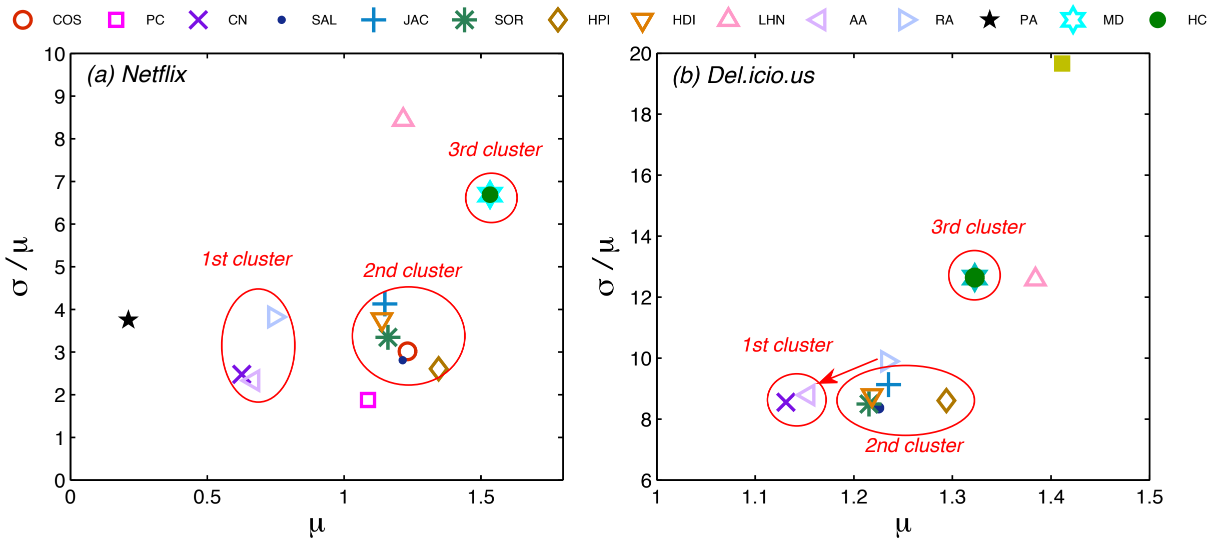

Fig. S2. The $\mu-\sigma$ location map with data amount parameter $\eta=0.5$ for Netflix and Del.icio.us dataset.

II. The top-n-stability method

As there are too many users (645056), objects (99622) and links (2036091) in the Amazon dataset, the calculation and memory requirement of recommending for Amazon data is out of our technological level. Thus, the stability of the recommendation is analyzed in five datasets, which is *MovieLens*, *Netflix*, *Last.FM*, *Epinions* and *Del.cio.us* respectively.

Table S1. Average ranking positions $<R>$ of 15 similarity indexes. Six most unstable indexes in each dataset are marked as bold red values.

	MovieLens	*Netflix*	*Last.FM*	*Epinions*	*Del.icio.us*
**COS**	0.02525	0.04523	—	**0.404167**	—
**PC**	**0.308995**	**0.155155**	—	0.298478	—
**CN**	0.00466	0.0082	0.01546	0.219239	0.126605
**SAL**	**0.10204**	**0.174705**	**0.249765**	**0.398832**	0.124205
**JAC**	0.026565	0.039805	**0.09492**	0.369603	0.12714
**SOR**	0.02665	0.03889	0.0792	0.3617	0.12616
**HPI**	**0.385435**	**0.424375**	**0.344195**	**0.45627**	**0.12952**
**HDI**	0.02755	0.037145	0.08336	0.344709	**0.129145**
**LHN**	**0.366225**	**0.4284**	**0.319725**	**0.45606**	0.12662
**AA**	0.004725	0.008355	0.01491	0.223996	**0.128695**
**RA**	0.004745	0.009815	0.016065	0.279887	**0.128865**
**PA**	0.003715	0.00427	0.003155	0.001342	0.0303
**MD**	0.00505	0.01374	0.037865	0.304379	**0.130675**
**HC**	**0.43738**	**0.448985**	**0.35846**	**0.430223**	**0.13509**
**IHC**	**0.440085**	**0.421155**	**0.319375**	**0.423226**	0.127185

The average ranking position $<R>$ is defined in the original paper to evaluate the recommendations lists' coherence, i.e., the stability of recommendation result. There is a classical metric evaluating the accuracy of the recommendations named *ranking score*, which is similar with our average ranking position $<R>$. However, one can find that, ranking score is to measure the accuracy of the recommendation and the average ranking position is to measure the stability of the recommendation lists. While ranking score measures the positions of users' real-selected objects, the average ranking position only measures the positions of objects that are recommended. Table S1 shows the results of $<R>$ when all the data is used. As has been discussed in the original paper, the average ranking position $<R>$ of the totally random case is 0.5 theoretically. Surprisingly, the average ranking positions $<R>$ of several indexes such as PC, SAL, HPI, LHN, HC and IHC may be over 0.1 or even over 0.4, which are very unstable results. On the other hand, some of the indexes such as CN, AA, RA and PA have very small values of $<R>$.

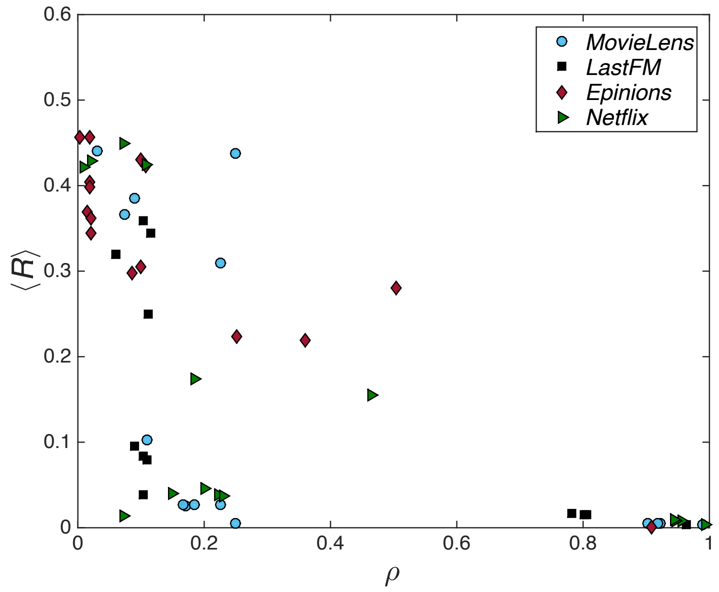

Fig. S3 The average ranking position $\langle R\rangle$ versus the Pearson coefficient of similarity matrix ρ for each similarity measurement. The values are calculated with $\eta = 0.5$ and $L = 50$ and averaged over 10 independent calculations.

To improve the stability of the recommendation, especially for the unstable indexes, the top-n-stability method is introduced in the original paper. Figure S4 reports the results of top-n-stability method for the Del.icio.us dataset. As the similarity stabilities of those indexes are similar with each other as shown in Fig. S1 and Fig. S2, the recommendation stabilities thus have no apparent difference. On can also find from Table S1 that, the recommendation stabilities in the Del.icio.us dataset are poor (average ranking positions are all larger than 0.1 except PA index). Thus, the improvements for each index are apparent, and only 1%~3% of the stable similarities are needed to get stable recommendation. The PA index is a special one that exhibits almost opposite pattern against the top-n-stability method. For every dataset, the more data been provided, the more stable the PA index would be.

In the original paper, we show the results of top-n-stability method according to the classification of indexes. There are four indexes' (PC, LHN, PA and IHC) results are not in Fig. 5 of the original paper and thusly we show here in Fig. S5. While the PA index could always generate stable recommendation, the stability of PC, LHN and IHC could be largely improved by the top-n-stability method.

Furthermore, the optimized ratios of stable objects, the optimized stabilities and the improved ratios of 15 similarity indexes are summarized in Table S2.

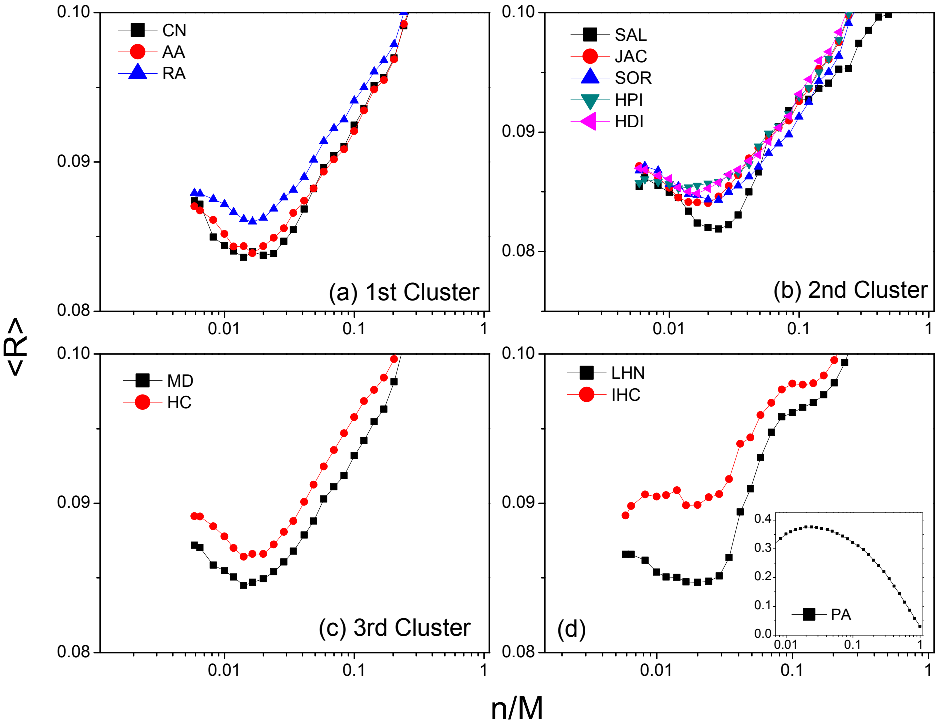

Fig. S4. The average ranking position $<R>$ of the top-n-stability method for the Del.icio.us dataset.

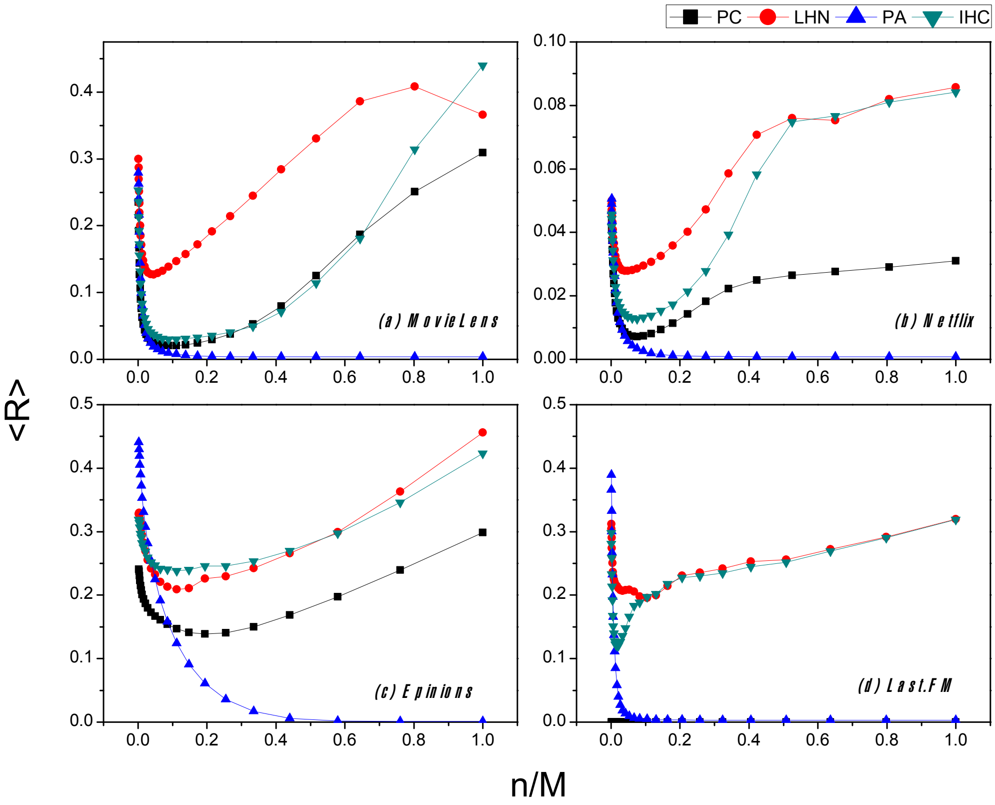

Fig. S5. Results of the top-n-stability method for the PC, LHN, PA and IHC index in the MovieLens (a), Netflix (b), Epinions (c) and Last.FM (d) respecitively.

Table S2. Stability results of the top-n-stability method. $n/M(opt.)$ means the ratio of table objects counted to get the most stable recommendation, R^{opt} means the most stable (small) value of the recommendation for the corresponding index, and R^{all} is the average ranking position when all the similarities are counted.

	MovieLens			Netflix			Last.FM			Epinions			Del.icio.us		
	$n/M(opt.)$	R^{opt}	R^{opt}/R^{all}	$n/M(opt.)$	R^{opt}	R^{opt}/R^{all}	$n/M(opt.)$	R^{opt}	R^{opt}/R^{all}	$n/M(opt.)$	R^{opt}	R^{opt}/R^{all}	$n/M(opt.)$	R^{opt}	R^{opt}/R^{all}
**COS**	0.1111	0.0099	0.3921	0.0941	0.0223	0.4937	—	—	—	0.1123	0.1746	0.4319	—	—	—
**PC**	0.0890	0.0207	0.0668	0.0758	0.0357	0.2303	—	—	—	0.1940	0.1386	0.4645	—	—	—
**CN**	0.4151	0.0046	0.9957	0.2753	0.0081	0.9902	0.7980	0.0146	0.9447	0.2550	0.1489	0.6794	0.0141	0.0836	0.6603
**SAL**	0.0890	0.0119	0.1164	0.0612	0.0342	0.1957	0.0214	0.0479	0.1919	0.0855	0.1685	0.4225	0.0241	0.0819	0.6592
**JAC**	0.1111	0.0118	0.4436	0.1165	0.0264	0.6622	0.0834	0.0401	0.4220	0.1123	0.1828	0.4947	0.0200	0.0841	0.6611
**SOR**	0.1111	0.0118	0.4439	0.1165	0.0262	0.6747	0.1046	0.0360	0.4548	0.1123	0.1790	0.4948	0.0241	0.0843	0.6683
**HPI**	0.0890	0.0109	0.0282	0.0492	0.0334	0.0788	0.0171	0.0596	0.1732	0.0647	0.1889	0.4141	0.0118	0.0853	0.6586
**HDI**	0.0890	0.0132	0.4804	0.1165	0.0270	0.7280	0.0834	0.0366	0.4392	0.1476	0.1788	0.5188	0.0165	0.0849	0.6572
**LHN**	0.0460	0.1269	0.3464	0.0398	0.1394	0.3253	0.1046	0.1955	0.6114	0.1123	0.2093	0.4589	0.0200	0.0847	0.6690
**AA**	0.2675	0.0047	0.9947	0.2222	0.0082	0.9856	0.7980	0.0141	0.9450	0.1940	0.1487	0.6638	0.0165	0.0839	0.6518
**RA**	0.4151	0.0047	0.9916	0.2753	0.0096	0.9776	0.7980	0.0153	0.9521	0.1476	0.1827	0.6526	0.0165	0.0860	0.6673
**PA**	1.0000	0.0037	1.0000	1.0000	0.0043	1.0000	1.0000	0.0032	1.0000	1.0000	0.0013	1.0000	1.0000	0.0303	1.0000
**MD**	0.3333	0.0049	0.9713	0.2222	0.0115	0.8333	0.1046	0.0227	0.6003	0.1476	0.1892	0.6216	0.0141	0.0845	0.6467
**HC**	0.0890	0.0213	0.0488	0.0612	0.0562	0.1251	0.0171	0.1136	0.3168	0.0004	0.2080	0.4834	0.0141	0.0864	0.6398
**IHC**	0.1111	0.0300	0.0682	0.0758	0.0641	0.1521	0.0171	0.1202	0.3764	0.0004	0.2091	0.4940	0.0059	0.0892	0.7012

III. The top-n-similarity method

The classical method named top-n-similarity that only takes the highest similarities into account when making recommendation, is very similar with our top-n-stability method. Our method focuses on the stability problem of the recommendation and aims to improve those unstable indexes' recommendation stability. To explore the difference between the top-n-stability method and the top-n-similarity method, we take MovieLens data as an example and show the comparison of the average ranking positions of those two methods in Fig. S6. Despite that, the stable indexes such as CN, AA, RA and PA exhibit similar results for those two methods, the top-n-stability method could better improve the recommendation stabilities of other indexes.

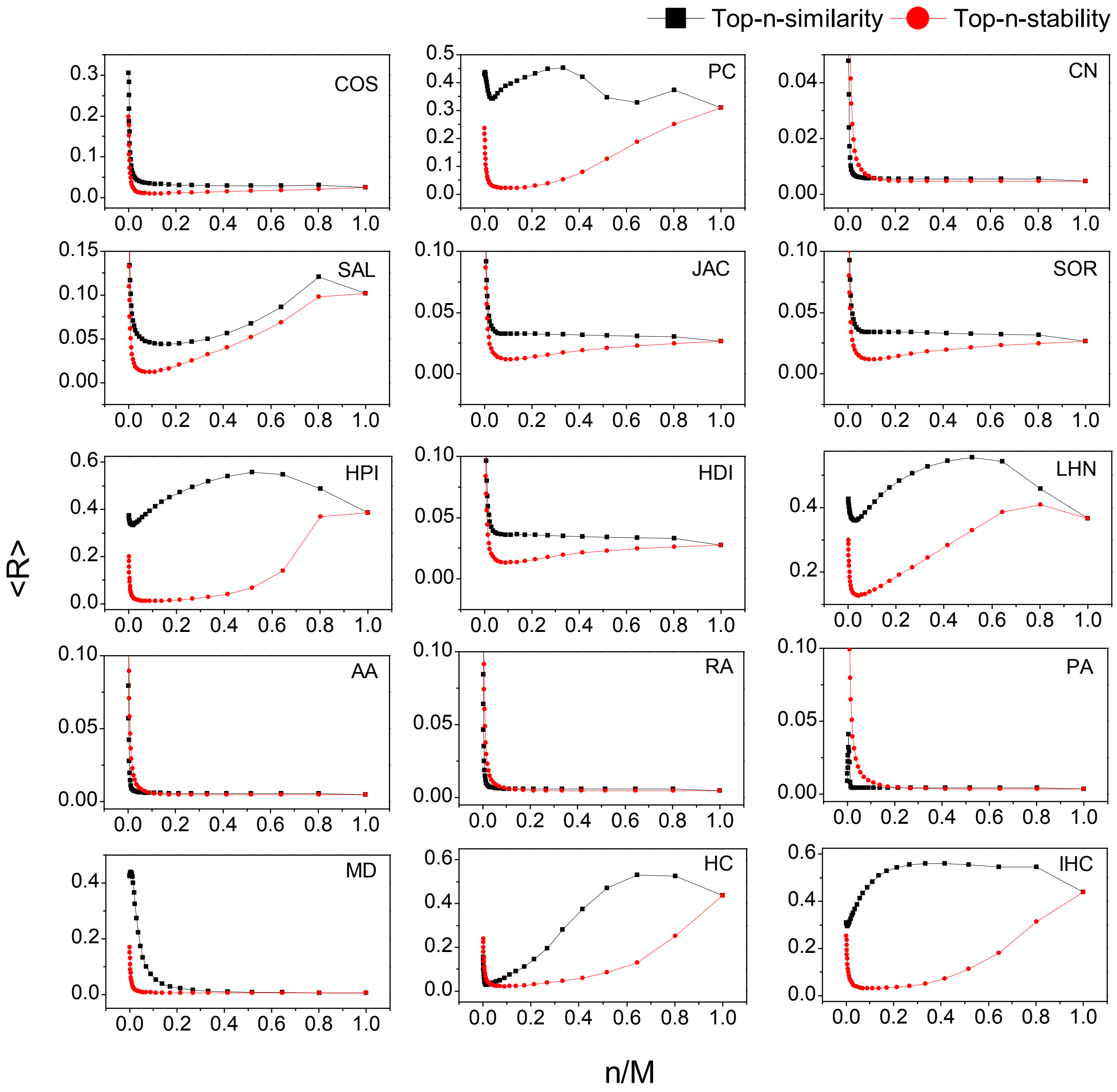

Fig. S6. Comparison of average ranking position between top-n-stability method and top-n-similarity method.

IV. Toy Dataset Test

1) Reshuffled Dataset

The reshuffled dataset may remain the degree distributions of both user and object, but replace the behavior-driven structure with random structures. Here we only take MovieLens as an example to explore the stability in reshuffled dataset. As stated in the main article, the measurements in the same cluster tend to have similar locations on the $\mu - \sigma$ map. Actually, as illustrated by the comparison between stability of empirical and reshuffled dataset shown in Fig. S7, the removing of user behavior's influence does not change the stability pattern of each measurement. Furthermore, as shown in Fig. S8, the measurements in the same cluster also share same stability pattern against data amount. The 1st cluster is sensitive when the data amount is little and rapidly increases to a stable state as the data become abundant. The similarity stability of measurements in the 2nd and 3rd cluster approximately follows the linear pattern against the data amount.

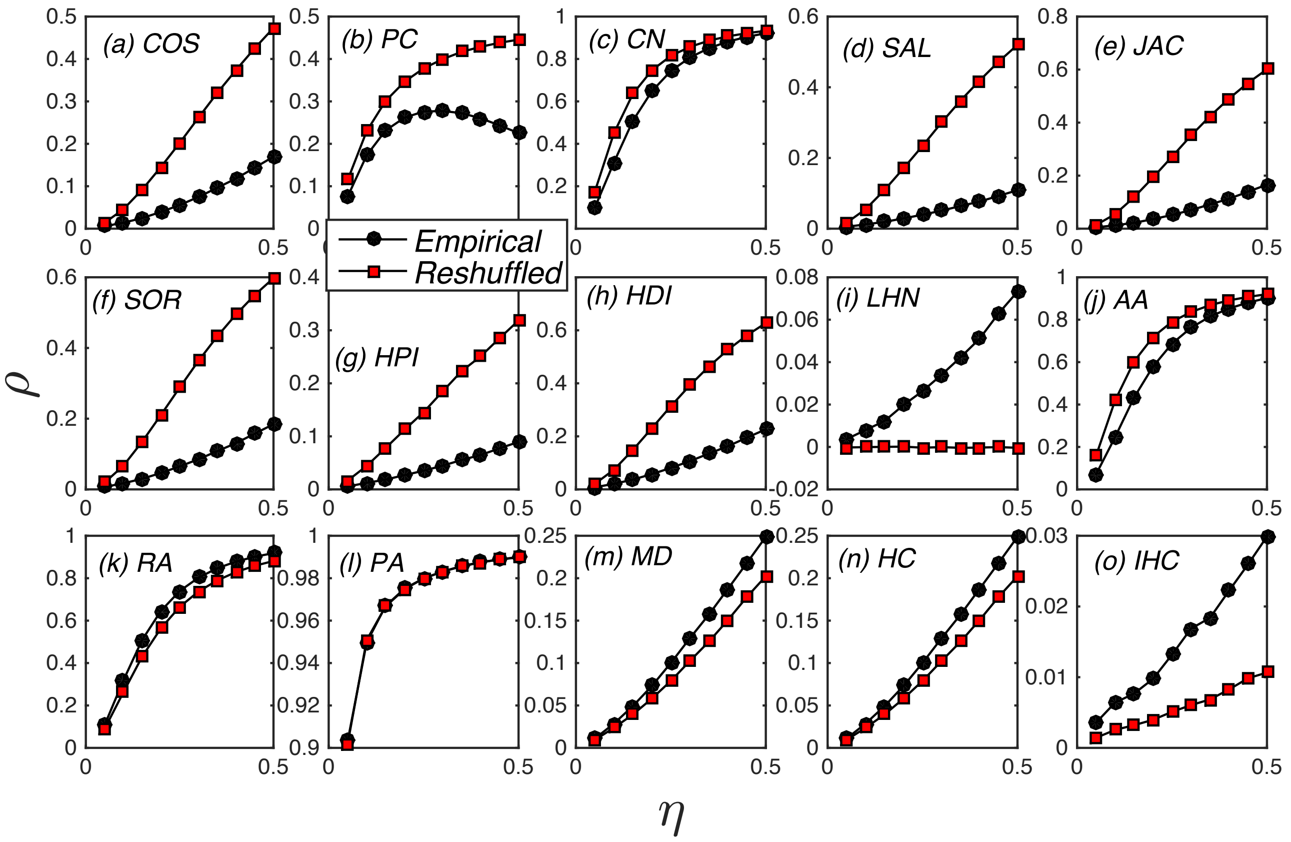

Fig. S7 Comparison of similarity stability between empirical and reshuffled MovieLens dataset.

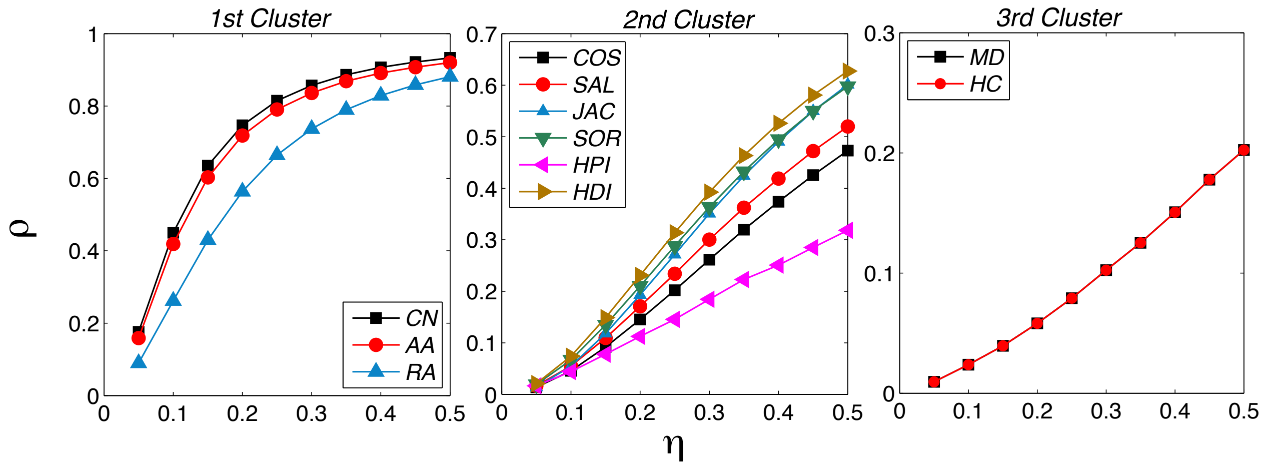

Fig. S8 Similarity stability ρ versus data amount parameter η for reshuffled MovieLens dataset.

2) Random Generated Dataset

In the generation of random dataset, we introduced the preferential mechanism to make the degree of both user and object power-law distributed as shown in Fig. S9. So, this random generated dataset would have similar degree distribution with the empirical dataset.

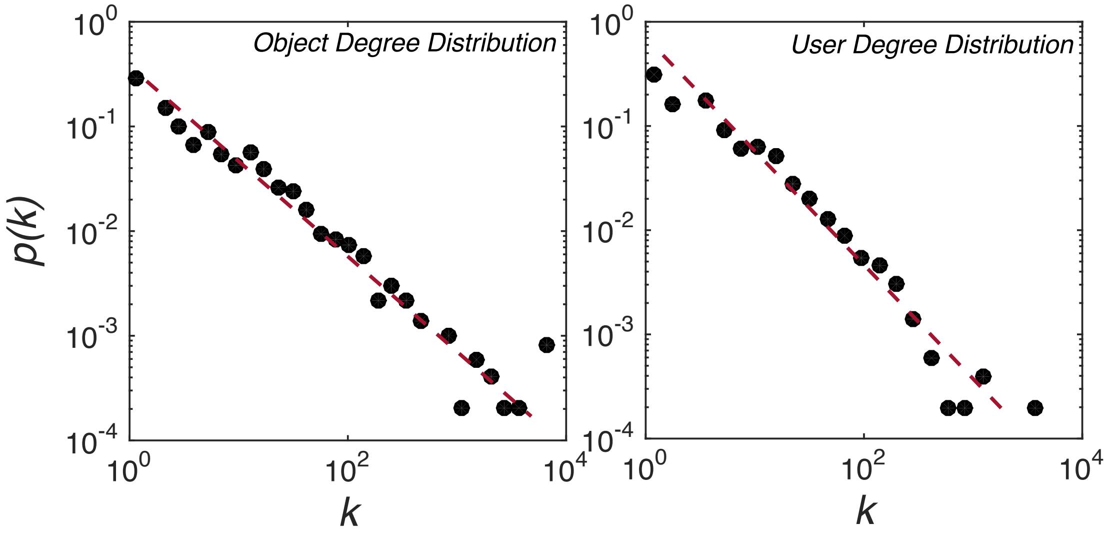

Fig. S9 Binned degree distributions of random generated dataset with number of objects and users of $M = N = 5000$, number of total links of $T = 10^5$ and the degree preference parameter $\gamma = 1.4$ (see the random data generating process in the main article). In each subplot, the binned distribution shown as black circles are calculated by dividing the degrees in to logistic bins.

As we could control the structure of the random generated datasets, we explore more about the influence of the structure on the similarity stability. Here we propose three schemes to study it. Scheme 1 is to fix the number of objects as $M = 5000$ and the average degree of objects as $\langle k_o \rangle = 20$, and gradually change the number of users. Under this scheme, as shown in Fig. S10, either too many users or too little users would lead to bad stability for most of the measurements. The optimized ratio of number of users and objects for similarity stability is 1:1 if the average object degree remains

unchanged. With the settings of scheme 1, users need to share finite links to users and thusly, when there are too many users, the dataset becomes very sparse. As to the case when there are little users, it is difficult for the system to determine the similarity between objects because it lack of common users. As an opposite case, scheme 2 is to fix the average degree of users, i.e. more users would bring more links to objects. Under this scheme, the similarity would be more stable when there are more users because they may bring more information. Furthermore, scheme 3 fixes the number of both users and objects as $M = N = 5000$, and increases the total links i.e. the average degree. In this case, more links bring more information and thusly, the similarities would be more stable.

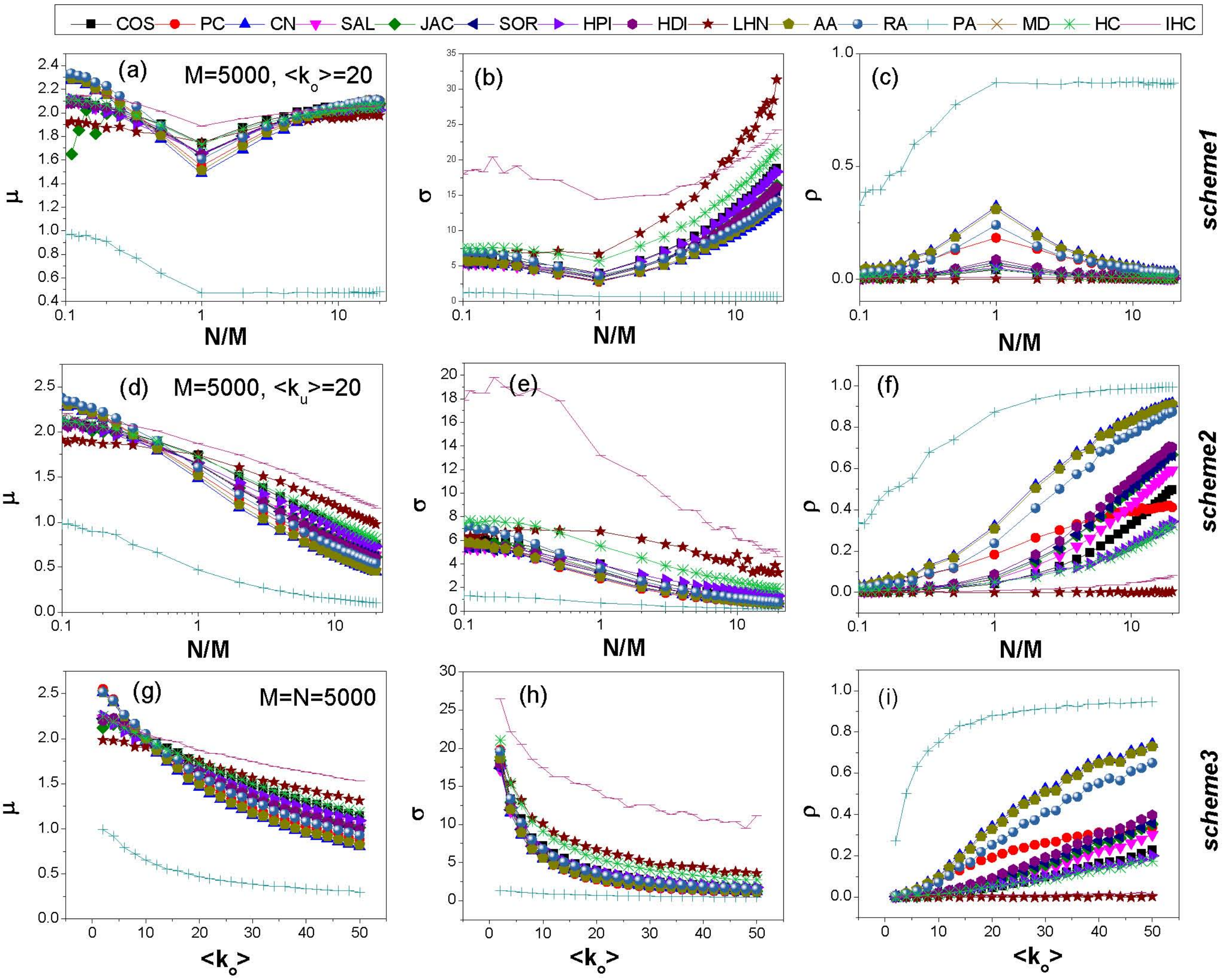

Fig. S10 Similarity stability for random generated dataset with different parameters.
